# The Phenotypic and Genotypic Features of *ADAMTSL4*‐Related Ocular Disease

**DOI:** 10.1111/cge.70109

**Published:** 2025-11-17

**Authors:** Katie M. Williams, Wolfgang Berger, Samuel Koller, Fatma Kivrak Pfiffner, Alessandro Maspoli, Jiradet Gloggnitzer, Britta V. T. Brühwiler, Christina Stathopoulos, Francis Munier, Louise Allen, Christos Iosifidis, Graeme C. Black, Panagiotis I. Sergouniotis, Ian Christopher Lloyd, Christina Gerth‐Kahlert

**Affiliations:** ^1^ Department of Ophthalmology Sight and Sound Centre, Great Ormond Street Hospital London UK; ^2^ Moorfields Eye Hospital NHS Foundation Trust London UK; ^3^ Section of Ophthalmology King's College London, St Thomas Hospital London UK; ^4^ Institute of Medical Molecular Genetics University of Zurich Schlieren Switzerland; ^5^ Zurich Center for Integrative Human Physiology (ZIHP) University of Zurich Zurich Switzerland; ^6^ Neuroscience Center Zurich (ZNZ) University and ETH Zurich Switzerland; ^7^ Department of Ophthalmology University Hospital and University of Zurich Zurich Switzerland; ^8^ Jules‐Gonin Eye Hospital Lausanne Switzerland; ^9^ Cambridge University Hospital NHS Trust Cambridge UK; ^10^ Division of Evolution, Infection and Genomics, School of Biological Sciences, Faculty of Biology, Medicine and Health University of Manchester Manchester UK; ^11^ Manchester Royal Eye Hospital and St Mary's Hospital Manchester University NHS Foundation Trust Manchester UK

**Keywords:** *ADAMTSL4*, ectopia lentis, ectopia lentis et pupillae, lens subluxation, spherophakia

## Abstract

Pathogenic variants in *ADAMTSL4* are an important cause of isolated ectopia lentis with an increasing number of genetically confirmed cases internationally. We sought to better describe ocular features seen with pathogenic variants in *ADAMTSL4*. We performed a retrospective, multicenter study examining the phenotypic and genotypic spectrum of *ADAMTSL4*‐associated ocular disease. We identified 41 individuals from 32 families with genetically confirmed *ADAMTSL4*‐related disease across six tertiary referral centers across Europe. Identified participants had a young age of diagnosis (median 1.3 years) and a highly myopic refractive error (mean SE −10.27 D). A diagnosis of ectopia lentis et pupillae was made in a third of cases, with a younger age at diagnosis (median 0.5 years). Subluxation tended to be in the inferior direction (~33%). Zonules were noted to be missing or absent in the majority of cases. Sixteen different pathogenic variants in *ADAMTSL4* were reported. A previously reported 20‐bp deletion (c.767_786del) was highly prevalent in this cohort (23/32), and all ectopia lentis et pupillae cases carried this variant. *ADAMTSL4*‐related disease tends to present at a younger age and be associated with higher myopia than other forms of ectopia lentis (such as *FBN1*). Early identification of typical phenotypic features alongside genetic testing can aid early, precise diagnosis and prevent unnecessary investigations.

## Graphical abstract

*ADAMTSL4*‐related ocular disease tends to present at a younger age and be associated with higher myopia than other forms of ectopia lentis (such as *FBN1*). A previously reported 20‐bp deletion (c.767_786del) was highly prevalent in this cohort (23/32), and all ectopia lentis et pupillae cases carried this variant.

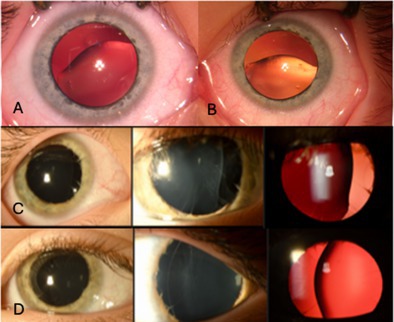

## 1 Introduction

Ectopia lentis is the dislocation or subluxation of the natural crystalline lens. This phenotype is typically associated with disorders disrupting the microfibrils of the ciliary zonules and results in instability or loss of zonule support. Nontraumatic ectopia lentis can occur as an isolated ocular disease or as part of a multisystemic condition. Multisystemic conditions such as Marfan syndrome (*FBN1*‐related disease) and homocystinuria can be implicated and prompt investigations, such as a cardiological evaluation and serum blood tests, are typically undertaken. However, genetic testing has been shown to have a high diagnostic yield and is increasingly used as a primary investigation [1, 2, 3]. Abnormalities in a number of genes have been implicated in the development of ectopia lentis, including *AASS*, *ADAMTS10*, *ADAMTS17*, *ADAMTSL4*, *ASPH*, *BCOR*, *CBS*, *COL18A1*, *FBN1*, *LTBP2*, *P3H2*, *PORCN*, *SUOX*, and *VSX2* [1].

Isolated (or “simple”) ectopia lentis is a group of phenotypes that are largely confined to the eye and may present soon after birth or later in life. The primary impact is on the lens, but other ocular anterior segment abnormalities may also be noted. *ADAMTSL4*‐related ectopia lentis appears to be the commonest reported genetic cause of isolated ectopia lentis (Gene ID: 54507; OMIM 610113) [4]. *ADAMTSL4*‐related ectopia lentis is inherited in an autosomal recessive pattern. In isolated ectopia lentis, the proportion of those with a pathogenic variant in *ADAMTSL4* appears to be around 50% in groups of individuals with White European ancestry [2, 5, 6, 7]. A range of ocular phenotypes have been reported in *ADAMTSL4*‐associated ectopia lentis, including ectopia lentis et pupillae, where the pupils are asymmetric, oval, and eccentric with pupil displacement in the opposite direction to a lens dislocation. Other reported structural abnormalities include congenital iris abnormalities such as iris hypoplasia, persistent pupillary membrane, and spherophakia. Secondary ocular features, likely related to the structural anomalies, include elevated intraocular pressure (IOP), retinal detachment, and high axial myopia [4]. The observed myopia is likely to have both a lenticular and an axial component and has been reported to assist in differentiating *ADAMTSL4*‐related ectopia lentis from *FBN1*‐related ectopia lentis in one series [5].

The increasing awareness of *ADAMTSL4*‐related disease, together with the more widespread availability of genetic testing, formed this international collaboration to provide more insight into this anterior segment disease. Phenotypic features were scrutinized, and associations between different affected variants and families were examined.

## 2 Materials and Methods

### 2.1 Study Populations

Study participants were retrospectively collected following a molecular diagnosis of *ADAMTSL4*‐related ocular disease. A multicenter, international case series was developed through collaborative links. Data were obtained from six tertiary referral ophthalmic centers. Inclusion criteria included: a genetic diagnosis of *ADAMTSL4*‐related ocular disease and availability of associated phenotypic description. We did not apply an age at onset or diagnosis as an exclusion criterion. Ophthalmic clinical details were systematically obtained from the participants' ophthalmologist, together with any additional systemic features or relevant evaluations. The results of genetic testing were similarly obtained from the participants referring to a tertiary medical center, with contact made where needed with the local genetic laboratory or geneticist.

Phenotypic data sought included demographics, family history, age at diagnosis/presentation, anterior segment features including zonule appearance, preoperative refractive error, and presence of preoperative IOP elevation and/or glaucoma. Surgical management, when performed, was recorded, and postoperative outcomes, including visual outcome and postoperative issues with IOP. Extraocular features, where present, were also recorded. The genetic and, to an extent, the clinical findings from seven affected probands have been previously reported [1]. The research adheres to the tenets of the Declaration of Helsinki and all patients consented to genetic testing. Demographic frequency features are presented by individuals affected or families affected. Ocular findings are presented as the frequency of affected eyes. Statistical differences between phenotypic groups were examined using the ANOVA test on Stata (StataCorp. 2025. *Stata Statistical Software: Release 19*. College Station, TX: StataCorp LLC.).

### 2.2 Genetic Testing

All participants underwent genetic testing via local genetic laboratories. Genomic analysis performed varied but included clinical exome sequencing, whole genome sequencing, and/or panel‐based analysis, and Sanger sequencing. The panel of genetic tests performed for participants from the United Kingdom and Switzerland is included in Appendix SA—importantly, the presence of ectopia lentis secondary to *FBN1* was tested. Clinical genetic testing was performed with the involvement of the clinical genetic teams at all clinical sites—bioinformatic support was used for variant analysis for the panels of genes tested and confirmed against known pathogenic variants. In a small subset, full panel genetic testing was not performed, but there were no clinical features suggestive of Marfan syndrome. All variants were reported on the GRCh37 reference genome. Interpretation of pathogenicity of variants identified was classified using four databases (Franklin, ACMG, ClinVar, and LOVD) using standard nomenclature.

## 3 Results

Overall, 41 individuals from 32 families met the inclusion criteria for this study (Tables 1 and 2). A male predominance was observed (*n* = 25, 61%). The majority of individuals were presumed to be of White European ancestry; however, 12.5% of families had South Asian (including Asian Pakistani) ancestry. The median age at diagnosis was 1.34 years, ranging from the first month of life up to 26 years. The majority (87%) were diagnosed before the age of 6 years. Pedigrees of the families are presented in Appendix SB; unfortunately, detailed pedigree information from some centers was not available.

**TABLE 1:** Phenotypic features of cases of *ADAMTSL4*‐related ocular disease.

ID	Age at diagnosis (years)	Pre‐op mean SE (D)	Pre‐op absolute mean astig (D)	Pre‐op RE astig (D)	Pre‐op LE astig (D)	Ectopia lentis et pupillae (+/−)	Spherophakia (+/−)	Zonule appearance	PPM (+/−)	Lensectomy	Lensectomy mean age (years)	Age at last exam (years)	RVA at last exam (LogMar)	LVA at last exam (LogMar)
1	NA	−5.50	3.00	−3.00	NA	−	+	NA	NA	RE	5	11	0	0
2	NA	0.19	NA	NA	NA	−	+	NA	NA	No	—	13	0	0
3	0	−5.50	1	NA	−1	+	Suspected	NA	NA	LE	2	10	0.2	0.7
4	0	−11.50	NA	NA	NA	+	Suspected	NA	NA	BE	1.5	12	0	0
5	0.3	−4.00	5.00	−5.00	−5.00	−	−	NA	NA	BE	5	8	0	0
6	0.5	−12.94	10.01	−10.25	−10.00	+ (LE)	+ (RE)	Absent nasally LE	NA	LE	0.5	2.5		
7	0	−15.06	4.13	−5.50	−2.75	+	+	Absent BE	NA	BE	2	3	0.6	0.7
8	0	−14.00	8.00	NA	−8.00	+	+	Only few visible BE	NA	BE	2	3	0.6	0.6
9	0.5	−0.25	6.00	5.00	−7.00	+	−	Absent LE	NA	LE	1.4	1.5	0.4	0.5
10	2.3	−17.50	2.00	−2.00	−2.00	−	+	NA	NA	No		2.5	0.8	0.5
11	3	−13.06	4.88	−6.00	−3.75	−	+	NA	NA	BE	3.7	24	1	0.8
12	0.4	−9.88	9.50	−8.75	−10.25	−	−	NA	NA	BE	0.6	11	1	1
13	1.2	−2.50	3.00	−4.50	−1.50	−	−	Partially absent BE	+	No	—	8	0.8	0.8
14	0.8	−7.31	4.88	−4.75	−5.00	+ (one eye)	−	NA	NA	BE	1	8	1	1
15	0.5	−6.44	2.38	−2.50	−2.25	+	−	NA	+ (LE)	BE	2	6	0.6	0.8
16	0.5	NA	NA	NA	NA	−	Suspected	NA	NA	LE	6.5	36	0.9	0.9
17	0.5	NA	NA	NA	NA	−	+	NA	NA	BE	3	37	0.8	1
18	1.7	−20.13	4.75	−6.00	−3.50	−	+	NA	NA	RE	20	29	0.8	0.5
19	4	−6.63	6.00	−8.50	−3.50	−	−	Partially absent BE	NA	RE	4.5	10	1	1
20	0	−12.44	9.13	−8.75	−9.50	−	+	NA	NA	BE	4	15	1	1
21	6	−8.25	3.50	−1.25	−5.75	−	−	Woken/absent BE	NA	LE	8	14	0.3	1.5
22	12	−8.63	7.25	−9.50	−5.00	−	−	NA	NA	No	—	18	0.52	1.12
23	5	−12.13	3.25	−5.00	−1.50	−	−	Absent/loss inferotemporally LE	NA	RE	5	10	0.36	0.1
24	3	−4.94	1.88	−3.00	−0.75	+	−	Deficient inferiorly LE	NA	RE	3	7	0.32	0.32
25	1	−27.50	4.00	−4.00	−4.00	+	−	Superior zonule loss BE	NA	BE	4	7	0.28	0.4
26	1	−19.00	NA	NA	NA	+	−	NA	+	BE	2	4	1	1
27	1	4.75	2.00	−2.00	−2.00	−	−	“Destroyed” BE	NA	LE	7	11	0.1	0.7
28	2	−21.13	2.25	−2.50	−2.00	−	−	NA	NA	No	—	5	1	1
29	2	−7.75	7.00	−6.00	−8.00	−	−	Broken/absent BE	NA	BE	3	3	0.36	0.36
30	2	−29.50	NA	NA	NA	−	−	NA	+	BE	10	32	0.16	0.6
31	1.5	−15.75	2.00	−1.50	−2.50	−	−	NA	+ (360°)	BE	3		0.2	0.35
32	2	−12.25	6.00	−6.50	−5.50	−	+	Broken superiorly BE	+	RE	NA	5	0.75	0.125
33	1	2.00	0.00	0.00	0.00	+	−	NA	NA	BE	2	10	0.4	0.34
34	1	−8.38	5.25	5.50	5.00	−	−	NA	NA	BE	3	13	0.5	0.2
35	2	NA	NA	NA	NA	−	−	NA	NA	BE	3	14	0.2	0.42
36	3	NA	NA	NA	NA	−	−	NA	NA	No	—			
37	18	NA	NA	NA	NA	−	−	NA	NA	LE	18			
38	26	−14.00	NA	NA	NA	−	−	Nasal dialysis BE	NA	No	—	26	0.1	0.3
39	1	−9.94	1.88	2.00	1.75	−	−	Absent superiorly BE	NA	BE	8	26	2	0.18
40	12	NA	NA	NA	NA	−	−	NA	NA	BE	43	63	0.3	1
41	3	−5.44	5.88	9.25	2.50	+	−	NA	NA	No	—	9	1.3	0.15

Abbreviations: “−”, absent, age at diagnosis given in fractional years when available; “+”, present, age at diagnosis given in fractional years when available; astig, absolute astigmatism; BE, both eyes; D, dioptres; IOP, intraocular pressure; LE, left eye; LVA, left visual acuity; PPM, persistent pupillary membrane; RD, retinal detachment; RE, right eye; RVA, right visual acuity; SE, spherical equivalent.

**TABLE 2:** Genotypic features of cases of *ADAMTSL4*‐related ocular disease.

FID	ID	Family history	Sex	Ethnicity	Allele 1 cDNA	Allele 1 protein	ACMG/ClinVar/LOVD	Allele 2 cDNA	Allele 2 protein	ACMG/ClinVar/LOVD
1	1	+ (ID 2)^a^	M	White European (Swiss)	c.2270dup	Gly758Trpfs*59	Pathogenic	c.2724T>A	Cys908*	Pathogenic, likely pathogenic
1	2	+ (ID 1)^a^	F	White European (Swiss)	c.2270dup	Gly758Trpfs*59		c.2724T>A	Cys908*	
2	3	+ (ID 4)^a^	F	White European (German/Swiss)	c.885_886del	Gly296Glufs*4	Pathogenic, likely pathogenic	c.767_786del	Gln256Profs*38	Pathogenic
2	4	+ (ID 3)^a^	M	White European (German/Swiss)	c.885_886del	Gly296Glufs*4		c.767_786del	Gln256Profs*38	
3	5		M	White European (Swiss)	c.1265G>A	Cys422Tyr	VUS	c.2593C>T	Arg865Cys	Likely pathogenic
4	6		F	White European (Swiss)	c.2520dup	Cys841Leufs*59	Pathogenic, likely pathogenic	c.767_786del	Gln256Profs*38	Pathogenic
5	7	+ (ID 8)^a^	M	White European (Dutch/Hungary)	c.767_786del	Gln256Profs*38	Pathogenic	c.767_786del	Gln256Profs*38	Pathogenic
5	8	+ (ID 7)^a^	M	White European (Dutch/Hungary)	c.767_786del	Gln256Profs*38		c.767_786del	Gln256Profs*38	
6	9		M	White European (Swiss)	c.193C>T	Gln65*	Likely pathogenic	c.767_786del	Gln256Profs*38	Pathogenic
7	10		F	White European (Swiss)	c.767_786del	Gln256Profs*38	Pathogenic	c.2254C>T	Gln752*	Pathogenic
8	11		F	White European (Swiss)	c.767_786del	Gln256Profs*38	Pathogenic	c.2382 + 1G>A	Splicing/p.(?)	Pathogenic, likely pathogenic
9	12	+ (ID 13)^a^	M	White European (Swiss)	c.3162‐3163insT	Thr1055Tyrfs*44	VUS, likely pathogenic	c.767_786del	Gln256Profs*38	Pathogenic
9	13	+ (ID 12)^a^	M	White European (Swiss)	c.3162‐3163insT	Thr1055Tyrfs*44		c.767_786del	Gln256Profs*38	
10	14	+ (ID 15)^a^	M	White European (Swiss)	c.767_786del	Gln256Profs*38	Pathogenic	c.767_786del	Gln256Profs*38	Pathogenic
10	15	+ (ID 14)^a^	F	White European (Swiss)	c.767_786del	Gln256Profs*38		c.767_786del	Gln256Profs*38	
11	16	+	M	White European (Swiss)	c.767_786del	Gln256Profs*38	Pathogenic	c.2594G>A	Arg865His	Pathogenic, likely pathogenic
11	17	+	F	White European (Swiss)	c.767_786del	Gln256Profs*38		c.2594G>A	Arg865His	
12	18		F	White European (Swiss)	c.2594G>A	Arg865His	Pathogenic, likely pathogenic	c.2594G>A	Arg865His	Pathogenic, likely pathogenic
13	19		F	White European (Swiss)	c.767_786del	Gln256Profs*38	Pathogenic	c.902delC	Pro301Leufs*42	Pathogenic, likely pathogenic
14	20		F	White European (Swiss)	c.767_786del	Gln256Profs*38	Pathogenic	c.767_786del	Gln256Profs*38	Pathogenic
15	21		M	White European (British)	c.767_786del	Gln256Profs*38	Pathogenic	c.2757G>T	Trp919Cys	VUS
16	22		M	Asian Pakistani	c.2237G>A	Arg746His	Pathogenic	c.2237G>A	Arg746His	Pathogenic
17	23	Family history of “congenital cataracts”	M	White European (British)	c.767_786del	Gln256Profs*38	Pathogenic	c.3089‐1G>A	Splicing/p.(?)	Likely pathogenic
18	24		M	White European (British)	c.767_786del	Gln256Profs*38	Pathogenic	c.767_786del	Gln256Profs*38	Pathogenic
19	25		M	White European (British)	c.767_786del	Gln256Profs*38	Pathogenic	c.2663G>A	Gly888Glu	VUS, likely benign
20	26	+ (ID 28)^a^	M	White European (British)	c.767_786del	Gln256Profs*38	Pathogenic	c.767_786del	Gln256Profs*38	Pathogenic
21	27		M	White European (British)	c.767_786del	Gln256Profs*38	Pathogenic	c.767_786del	Gln256Profs*38	Pathogenic
20	28	+ (ID 26)^a^	M	White European (British)	c.767_786del	Gln256Profs*38		c.767_786del	Gln256Profs*38	
22	29		F	White European (British)	c.421C>T	Arg141*	Pathogenic	c.2305C>T	Leu769Phe	VUS, likely benign
23	30		M	Asian Pakistani	c.767_786del	Gln256Profs*38	Pathogenic	c.767_786del	Gln256Profs*38	Pathogenic
24	31		F	South Asian (Indian)	c.376C>T	p.(Arg126Ter)	Pathogenic	c.2021_2022del	p.(Ser674CysfsTer21)	Pathogenic
25	32		F	White European (French)	c.767_786del	Gln256Profs*38	Pathogenic	c.767_786del	Gln256Profs*38	Pathogenic
26	33		M	White European (British)	c.767_786del	Gln256Profs*38	Pathogenic	c.767_786del	Gln256Profs*38	Pathogenic
27	34		M	White European (British)	c.767_786del	Gln256Profs*38	Pathogenic	c.2236C>T	Arg746Cys	Likely pathogenic
28	35		F	Asian Pakistani	c.1234G>A	Val412lle	VUS	c.1234G>A	Val412lle	VUS
29	36	+ (mum ID 37 and maternal aunt ID 38)	M	Arabic	c.2237G>A	Arg746His	Pathogenic	c.2237G>A	Arg746His	Pathogenic
29	37	+ (son ID 36 and sister ID 38)	F	Arabic	c.2237G>A	Arg746His		c.2237G>A	Arg746His	
29	38	+ (sister ID 37 and nephew ID 36)	F	Arabic	c.2237G>A	Arg746His		c.2237G>A	Arg746His	
30	39		M	White European (British)	c.767_786del	Gln256Profs*38	Pathogenic	c.767_786del	Gln256Profs*38	Pathogenic
31	40		M	White European (British)	c.2236C>T	Arg746Cys	Likely pathogenic	c.2594G>A	Arg865His	Pathogenic, likely pathogenic
32	41		F	White European (Polish)	c.767_786del	Gln256Profs*38	Pathogenic	c.767_786del	Gln256Profs*38	Pathogenic

*Note*: Variant numbering on cDNA and protein levels refers to the MANE Select transcript NM_019032.6 (*ADAMTSL4*). All variants were classified as pathogenic or likely pathogenic according to ACMG (American College of Medical Genetics and Genomics) criteria and ClinVar and/or LOVD (Leiden Open Variation Database) entries except if indicated as VUS (variant of unknown significance) or likely benign.

^a^ Sibling affected.

### 3.1 Clinical Findings

**FIGURE 1:**
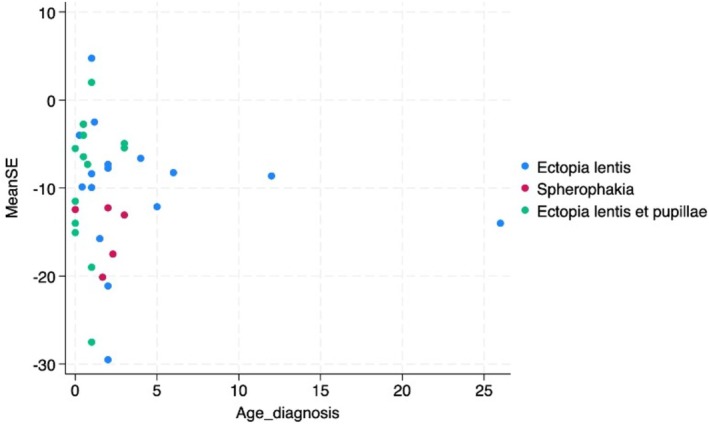
Scatter plots of mean spherical equivalent (mean SE) against age at diagnosis in cases of ectopia lentis alone, ectopia lentis et pupillae, and the presence of spherophakia.

Preoperative refraction was available for 36 individuals. There was a highly myopic spherical equivalent (SE) across the case series. The mean SE in both eyes was −10.27 (SD 7.49). The distribution of SE against age at presentation is illustrated in Figure 1. There was a high degree of astigmatism and an absolute astigmatism of 4 D was documented in 58% of eyes with ectopia lentis.

The direction of subluxation has been traditionally used to provide etiological clues in ectopia lentis. However, this feature is highly heterogeneous amongst individuals with the same underlying genetic etiology [8]. In this case series of *ADAMTSL4*‐related ectopia lentis, the most common direction of subluxation was inferior (just over 1/3); however, all directions of subluxation were observed (Table 3).

**TABLE 3:** Direction of lens subluxation in *ADAMTSL4*‐related ectopia lentis (data available *n* = 36 eyes).

	No. of individuals (% total)		Notes
Superior	2	5 (14%)	Unilateral left eye superior deviation (*n* = 1)
Superotemporal	3		
Superonasal	2		
Inferior	6	12 (33%)	Unilateral left eye inferotemporal deviation (*n* = 1) and LE inferonasal deviation (*n* = 1)
Inferotemporal	5		
Inferonasal	1		
Nasal	5	5 (14%)	Unilateral left eye nasal deviation (*n* = 1)
Temporal	9	9 (25%)	
Asymmetric subluxation	3	3 (8%)	Inferior right and superior left eye (*n* = 1); inferonasal right and inferotemporal left eye (*n* = 1); nasal right and superior left (*n* = 1)

**FIGURE 2:**
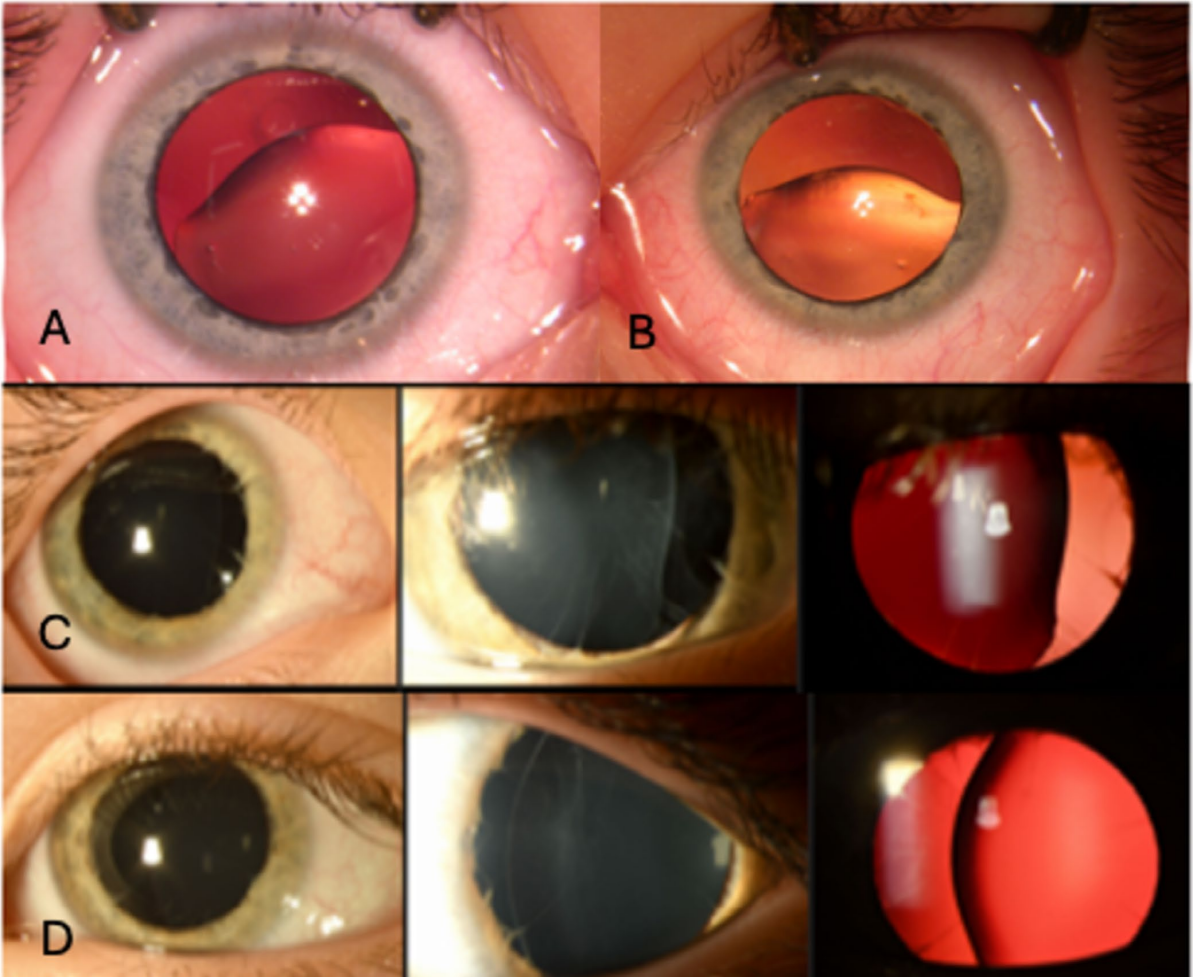
Anterior segment photos of two affected cases. Panel A illustrates right eye inferonasal lens subluxation with zonule absence and temporal notching of the peripheral lens as a result of residual zonule adhesion. In Panel B, the left eye of the same case shows inferior lens subluxation, absent zonules, and nasal peripheral lens deformation. Panel C illustrates the right eye of a second case with temporal subluxation and largely absent zonules, with the same features seen in the left eye of the same case in Panel D. In both cases, a degree of iris hypoplasia is seen.

Where documented, the zonule appearance was described as broken, absent, missing, or not visible in 28 eyes (4 unilateral cases and 12 bilateral cases). This was sometimes just one sector or a single “clock hour.” Notching of the lens border was noted in some cases. This was identified most clearly when intraoperative images and videos were obtained (as illustrated in Figure 2) and gave the lens an “anvil” shape (with residual zonules observed to pull at the points on the lens rim where they were still attached).

The presence of iris abnormalities such as hypoplasia and transillumination was not consistently recorded. The presence of a pupillary membrane was observed in six cases, but the documentation of this feature was limited, and we therefore suspect that this is an underestimate of this ocular feature (Table 2). One study participant was identified to have developed a retinal detachment in the left eye—this was a child who was diagnosed with isolated ectopia lentis, very high myopia (right SE −27 D, left SE −32 D), and underwent bilateral lensectomy at the age of 10 years.

### 3.2 Ectopia Lentis et Pupillae

As detailed in Table 1, in 13 study subjects, a diagnosis of ectopia lentis et pupillae was made (32% of individuals, 34% of families), which was bilateral in 85% of cases (*n* = 11). There was a male predominance (69%). The median age at diagnosis was younger than the overall cohort at 0.50 years (range, first month of life up to 3 years). The individuals were all of presumed White European ancestry. High myopia was again common with a mean SE between the two eyes of −9.84 D (SD 7.98). Absolute astigmatism higher than 4 D was documented in 55% of eyes.

### 3.3 Spherophakia

A diagnosis of spherophakia was noted in eight study subjects (91% bilaterally), whilst it was suspected in an additional three patients. In those confirmed, there was a female predominance (73%), and the median age at diagnosis was again younger than the overall cohort (0.50 years, ranging from the first month of life up to 3 years). All individuals were of presumed White European ancestry. Greater degrees of myopic refractive error (mean SE −11.53, SD 6.91) were observed. Absolute astigmatism higher than 4 D was documented in 57% of eyes.

At presentation, one case was noted to have elevated IOP (IOPs right 27 mmHg, left 29 mmHg). The case was a male of presumed White European ancestry diagnosed with isolated ectopia lentis (with bilateral superotemporal lens subluxation) at the age of 1 year. The boy underwent bilateral lensectomy at the age of 3 years and there was no IOP elevation postoperatively.

Lensectomy was performed in 81% of cases (34/42), of which 62% (21/34) underwent bilateral surgery. The median age at surgery was 3.0 years, with a range from 6 months to 43 years. Surgery was performed at a younger age in the cases of ectopia lentis et pupillae (median 2 years) and spherophakia (median 4 years), compared to ectopia lentis alone (median age 5.8 years)—this trend was statistically significant (*p* < 0.01). All but three cases were left aphakic, the latter of whom included: one case who had subsequently undergone bilateral artisan IOL insertion for contact lens intolerance; one case who underwent iris clipped IOL insertion in one eye; and one who underwent bilateral IOL insertion at the time of surgery in adulthood, which subsequently dislocated and was replaced with secondary anterior chamber IOLs.

Following surgery, a visual acuity measurement was available for 31 of the 33 cases that underwent surgery. The median visual acuity in the better seeing eye following surgery was 0.33 LogMAR (range 0.00–1.00 LogMAR), compared to a median visual acuity in the better seeing eye of 0.50 LogMAR (range 0.00–1.00 LogMAR) in those who had not undergone surgery, although this trend was not statistically significant (*p* = 0.59).

Three patients (all male of presumed White European ancestry) were reported to have postoperative elevated IOP. The underlying diagnosis was unilateral ectopia lentis (1/3) or bilateral ectopia lentis and pupillae (2/3) with an age at surgery of 7, 1.5, and 2 years, respectively. Unilateral IOP elevations were documented in the latter study subject who had bilateral manifestations. Topical treatment alone was sufficient to achieve IOP control in all cases. The study subject diagnosed with elevated IOP preoperatively did not require further intervention, as IOP normalized in the postoperative period.

### 3.4 Genetic Findings

Genetic testing identified a wide range of known pathogenic variants in the *ADAMTSL4* gene, including frameshifting deletions, two single‐nucleotide duplications and one insertion, but also single‐nucleotide variants (SNVs) resulting in amino acid substitutions or affecting splice sites (Table 2). The most common are deletions, with compound heterozygous changes detected in 50% of cases. There was no consanguinity reported in the patients with homozygous mutations. However, we agree that specifically family 29 is highly suggestive of consanguinity within the family.

Out of 32 families, 23 families (72%) were found to carry c.767_786del, p.(Gln256Profs*38), a previously reported 20‐bp deletion in exon 6 (out of 19 exons in the MANE select transcript NM_019032.6) of the *ADAMTSL4* gene. Among these, 11 were homozygous (34%) and 12 were compound heterozygous (38%) with a different variant on the second allele of the *ADAMTSL4* gene.

The majority of individuals who carried the pathogenic c.767_786del variant had been diagnosed with ectopia lentis et pupillae (45%), and in fact, all cases of ectopia lentis et pupillae in this series carried the c.767_786del variant. Among individuals who were homozygous for the c.767_786del variant, 57% were identified to have ectopia lentis et pupillae and 29% had spherophakia. Of those who were carrying the c767_786del variant but who did not have ectopia lentis et pupillae, 33% had spherophakia.

We identified three families in which both included family members carrying the same compound heterozygous mutations and the same phenotype. These included fam ID 1 (c.2270dup; c.2724T>A) with two siblings presenting with spherophakia, fam ID 2 (c.885_886del; c.767_786del) with two presenting with ectopia lentis et pupillae and suspected spherophakia, and fam ID 9 (c.3162‐3163insT; c.767_786del) with two siblings presenting with ectopia lentis. This is also illustrated in the pedigrees in Appendix SB. We identified individuals from four families carrying the c.2594G>A variant—two siblings with compound heterozygous mutations (c.2594G>A; c.767_786del) who both had ectopia lentis and spherophakia, a singleton with a homozygous mutation who had ectopia lentis and spherophakia, and a singleton with a compound heterozygous mutation (c.2594G>A; c.2236C>T) who had just ectopia lentis. We identified four individuals homozygous for the c.2237G>A variant, three individuals from the same family and one unrelated singleton, all of whom just had ectopia lentis. Finally, two unrelated individuals carried compound heterozygous mutations, including the c.2236C>T variant, both of whom had just ectopia lentis.

In addition to this frequent frameshift deletion, we detected four different nonsense variants, one single‐nucleotide insertion, one 2‐bp deletion, and two single‐nucleotide duplications in the protein‐coding sequence of the gene. The majority of pathogenic variants gave rise to premature termination codons (43/66 pathogenic alleles, 65%), which may lead to a truncated protein or nonsense‐mediated decay (NMD) of the *ADAMTSL4* mRNA. Amino acid substitutions appear to be less frequent. Two variants potentially affect the splicing of the *ADAMTSL4* mRNA.

In addition, 5 of the 23 detected variants were deemed to be variants of uncertain significance (VUS). In four patients, a VUS was combined with a pathogenic or likely pathogenic variant. However, one study participant was homozygous for a VUS. In this case, the phenotype was felt by the clinical team to be highly specific for *ADAMTSL4*‐related ocular disease, and testing had been performed to identify the majority of the alternative known genetic causes of the condition in question.

Before genetic diagnosis, many of the children underwent investigations such as a cardiac echo, given the possibility of underlying Marfan syndrome and/or serological testing to exclude homocystinuria. Systemic features reported in our series included one child with a closed meningocele, syndactyly, and small renal cysts, and a second with hypospadias.

## 4 Discussion

We report a case series of 41 individuals with *ADAMTSL4*‐related ocular disease. We identify a young age of onset (median 1.3 years), a male predominance, a highly myopic refractive error, and high degrees of astigmatism. In a third of cases, a diagnosis of ectopia lentis et pupillae was made, and individuals with this feature tended to have a younger age at diagnosis. A younger age of onset was also seen in the 26% that was diagnosed with spherophakia. *ADAMTSL4*‐related ectopia lentis tended to be inferior (~33%), but all directions of subluxation were observed. Zonules were noted to be missing or absent in the majority of cases, with a characteristic deformity on the lens rim where residual zonules were present.

We identified associated lens pathologies such as spherophakia in 19% of cases. This previously reported feature of *ADAMTSL4*‐related disease is likely to be related to the zonules not holding the lens in place and, with the elasticity of the lens, leading it to take on a “rounded” shape [9]. In this series, there were (i) two cases where just unilateral lens subluxation was noted; (ii) a further case diagnosed with ectopia lentis et pupillae in just one eye; and (iii) one individual who had a diagnosis of ectopia lentis et pupillae in one eye and spherophakia in the other. In a further three subjects, the direction of lens subluxation was different between the two eyes. Anisometropia (≥ 3 D) was very prevalent among our cases (*n* = 17), with many developing high anisometropia up to a maximum difference of 20.1 D in one case.

Lensectomy was performed in 81%. Surgery was performed at a younger age in the cases of ectopia lentis et pupillae (median 2 years) and spherophakia (median 4 years), compared to ectopia lentis alone (median age 5.8 years). The reported association of retinal detachment was seen in one eye of one child who had very significant myopia [9]. The rates of elevated IOP were low in our series—before surgery, just one case was affected, and post surgery, three cases had elevated IOP, with all responding to topical therapy. Two of these latter cases were children diagnosed with ectopia lentis et pupillae that had undergone surgery at a young age (1.5 and 2 years).

Extraocular findings in *ADAMTSL4*‐related ocular disease are uncommon, and as such, further investigation of systemic features is not warranted. However, there are some reports in the literature of features such as craniosynostosis, hernias, and tall stature [2, 10, 11, 12, 13]. The extraocular features reported in this cohort include one case with syndactyly, an anomaly previously reported in one case of *ADAMTSL4*‐related ocular disease [9].

*FNB1*‐related disease (including Marfan syndrome) represents a high proportion of genetically characterized ectopia lentis. Previous research has identified *ADAMTSL4*‐related ectopia lentis to have earlier identified ocular manifestations than *FBN1*‐related disease—the median age of diagnosis in one series of 17 patients was reported to be 2 years in the former compared to 35 years in the latter [5]. They also noted higher mean axial lengths in those with *ADAMTSL4*‐related ocular disease: 27.54 vs. 22.74 mm. Whilst we cannot report axial length measurements on all our cases, we did identify a similarly young median age at diagnosis (median 1.2 years). Highly myopic refractive errors, which we infer may in part be related to axial elongation, were also noted [14]. These findings are corroborated in a previous series of 12 patients with *ADAMTSL4*‐related disease, where all study subjects bar one were diagnosed in early childhood and where myopia exceeding −5 D was a common finding [9].

Subluxation and the altered shape of the crystalline lens are presumed to be the prime drivers for a myopic refractive error in these individuals. In addition, undercorrection of myopia (which is likely commonplace in these children, where accurate refraction is difficult) is a strong driver of axial elongation. However, it is interesting to note that alterations in *ADAMTSL4* have been implicated in the etiology of non‐syndromic myopia in the general population. When researchers performed gene‐based analyses, *ADAMTSL4* was enriched for genetic variants associated with refractive error in genome‐wide association analyses [15]. Gene expression of *ADAMTSL4* has been documented in the iris and choroidal tissue, suggesting further functional roles of this gene in the eye [16].

In this series, seven families had two affected family members, and one family had three affected family members. Among family members, the broad ophthalmic phenotype was very similar (all family members with ectopia lentis alone = 2, spherophakia *n* = 1, and ectopia lentis et pupillae *n* = 3). However, a degree of intrafamilial variability was noted in two families: one where only one sibling had a diagnosis of spherophakia, and another where only one sibling had a diagnosis of ectopia lentis et pupillae. The age at diagnosis between family members was comparable (mean difference 2.1 years, SD 5.24, paired *t*‐test = 0.30), as were the rates of lensectomy (all family members undergoing or not undergoing lensectomy in 55%) (Pearson *χ* ^2^ = 0.56) at similar ages (mean difference 0.4 years, standard deviation 3.07, *p* = 0.79).

A number of different pathogenic variants in *ADAMTSL4*‐related ectopia lentis have been reported, with the most common being c.767_786del [11, 12]. This variant was identified in 72% of families in this case series. This pathogenic 20‐bp deletion was detected in all cases of ectopia lentis et pupillae in this cohort. Individuals carrying this variant were significantly younger at diagnosis. The c.767_786del change is common in individuals of presumed White European ancestry and has been reported as a probable founder mutation, initially in a Norwegian population of ectopia lentis et pupillae [11, 12, 17, 18, 19]. A participant in this study carrying this deletion was of Asian Pakistani ancestry—however, the lack of targeted haplotypic analysis limits further comment on the potential founder mutation status of this variant.

Limitations of this study center on its retrospective nature, which limited the accurate identification of certain features, including iris anomalies, persistent pupillary membrane, and zonule appearance. In addition, a comprehensive assessment of extraocular features was not undertaken as the focus of this paper was the ophthalmic phenotype.

In conclusion, ectopia lentis associated with *ADAMTSL4*‐related disease tends to have a relatively early age of onset and a high myopic refractive error. Missing zonules are prevalent in our series and are likely the reason for abnormal lens shapes (such as the spheropheric and/or the “anvil” shape noted in some study subjects). Unsurprisingly, in cases of ectopic lentis et pupillae, the age at diagnosis and lensectomy was earlier. The rates of elevated IOP and retinal detachment were low. Improved understanding of the typical features of *ADAMTSL4*‐related oculopathy will aid in the individualization of further investigations and medical care.

## Conflicts of Interest

The authors declare no conflicts of interest.

## Supporting information


**Appendix SA:** Genes included in gene panels used for testing cases from the Switzerland and the UK.

**Appendix SB:** Family pedigrees.

## Data Availability

The data that support the findings of this study are available on request from the corresponding author. The data are not publicly available due to privacy or ethical restrictions.
