# Supplementary material for: The Phenotypic and Genotypic Features of ADAMTSL4‐Related Ocular Disease
Source: Clin Genet. 2025 Nov 17;109(4):730–41. doi: 10.1111/cge.70109 (PMC12958011; doi:10.1111/cge.70109)
Supplement: Supplementary file 1 — Appendix SA: Genes included in gene panels used for testing cases from the Switzerland and the UK. [file CGE-109-730-s001.pdf]

Switzerland panel of genes tested	UK panel and genes tested
AASS	ABCB6
ABCA3	ACTB
ABCB6	ACTG1
ABCD3	ADAMTS10
ABHD12	ADAMTS17
ADAM9	ADAMTS18
ADAMTS10	ADAMTSL4
ADAMTS17	ALDH1A3
ADAMTS18	ALX1
ADAMTSL4	ANK3
ADIPOQ	ARHGAP35
AGK	ASPH
ALDH18A1	ATOH7
ANK2	B3GALNT2
AP4B1	B3GLCT
APP	BCOR
ARID1B	BEST1
ARSL	BMP4
ASPH	BMPR1B
ATAD3A	C12orf57
B3GLCT	CAPN15
B4GALT7	CBS
BCOR	CC2D2A
BEST1	CDON
BFSP1	CENPF
BFSP2	CEP290
BIN3	CHD7
BRD4	CHRDL1
BUB1B	CLDN19
CANX	COL18A1
CAV1	COL4A1
CBS	CPAMD8
CC2D2A	CREBBP
CCNP	CRIM1
CDC25B	CRYAA
CHD7	CRYBB1
CHMP4B	CRYBB2
CLPB	CRYGC
CNBP	CYP1B1
CNGB3	DDX58
COL11A1	DOCK6
COL18A1	DYRK1A
COL2A1	EPHA2

COL4A1
COL4A2
COL4A5
COL7A1
CPAMD8
CRYAA
CRYAB
CRYBA1
CRYBA2
CRYBA4
CRYBB1
CRYBB2
CRYBB3
CRYGA
CRYGB
CRYGC
CRYGD
CRYGS
CRYZ
CTDP1
CTNND2
CYP27A1
CYP51A1
DHCR7
DMPK
DNASE2B
DNM2
DNMBP
DOCK5
DST
DYNC1H1
EFNA5
EIF2B2
EPG5
EPHA2
ERCC2
ERCC6
ERCC8
ESCO2
ETFDH
EYA1
EZR
FAM126A
FAR1

ESCO2
FAT1
FBN1
FKTN
FOXC1
FOXD3
FOXE3
FRAS1
FREM1
FREM2
FZD5
GDF6
GJA1
GJA8
GRIP1
HCCS
HHAT
HMX1
IFIH1
INPP5E
ISPD
KDM6A
KIAA0586
KIAA1109
KIF11
KMT2D
LAMB2
LMX1B
LRP2
LRP5
LTBP2
MAB21L2
MAF
MAPRE2
MFRP
MIR204
MITF
MYOC
MYRF
NAA10
NDP
NHS
NUP188
OCRL

FBN1
FKRP
FKTN
FLNB
FOXE3
FTL
FYCO1
FZD4
GALE
GALK1
GALT
GBA2
GCM2
GCNT2
GDF3
GEMIN4
GFER
GJA1
GJA3
GJA8
GJB6
GLA
GNAS
GNPAT
GPR161
GPX1
GSTM1
GSTT1
GTF2IRD1
GUCY2D
HCCS
HIP1
HMX1
HSF4
HSPG2
IARS2
IDO1
IFNGR1
IKBKG
INPP5K
INTS1
IPO13
ITM2B
JAM3
OFD1
OTX2
PACS1
PAX2
PAX6
PIGL
PITX2
PITX3
POMGNT1
POMT1
POMT2
PORCN
PRR12
PRSS56
PTCH1
PUF60
PXDN
RAB18
RAB3GAP1
RAB3GAP2
RARB
RAX
RBP4
RERE
RHOA
RIPK4
RPGRIP1L
SALL1
SALL4
SBF2
SH3PXD2B
SHH
SIX6
SLC25A24
SLC38A8
SLC4A4
SMCHD1
SMG8
SMO
SMOC1
SOX2
SRD5A3
STRA6
TBC1D20

KCNA4
KCNAB1
KCNJ13
KIAA1109
KLRG1
LARGE1
LCA5
LCT
LEMD2
LGSN
LIM2
LMX1B
LONP1
LOXL3
LRP2
LRP5
LRP5L
LSS
LTBP2
LTBP3
MAB21L2
MAF
MAFA
MAN2B1
MED12
MED13
MIP
MIPEP
MIR184
MMP1
MVK
MYH9
MYOC
NACC1
NAT8
NCOA6
NDP
NECAP2
NECTIN3
NEU1
NF2
NHS
NOD2
NR2E3
TEK
TENM3
TFAP2A
TMEM216
TMEM237
TMEM5
TMEM67
TMEM98
TUBGCP4
VSX2
WDR37
WLS
YAP1
ZEB2
AIPL1
BMP7
BMPR1A
BRPF1
C16orf62
CDH2
CDH4
CDK5RAP2
CNNM4
COL2A1
COL6A3
COX7B
CRB1
CRYBA4
CRYBB3
CRYGD
DAG1
EFTUD2
ERCC1
FANCL
FKRP
FZD4
GDF3
GLI2
HMGB3
IGBP1
IPO13
KERA
OLFM2
PDE6D

NRCAM
OAT
OCRL
OGG1
OPA1
OPA3
OTX2
P3H2
PANK4
PARK7
PAX6
PDE6B
PEX1
PEX10
PEX11B
PEX12
PEX13
PEX14
PEX16
PEX2
PEX26
PEX3
PEX5
PEX6
PEX7
PIGY
PITX2
PITX3
PKN1
PNPT1
POLG
POMGNT1
POMT1
POMT2
PORCN
PQBP1
PRDX5
PROX1
PRX
PTCH1
PTEN
PTH
PXDN
RAB3GAP1
PDGFRA
PLK4
POMGNT2
PQBP1
SALL2
SCLT1
SIX3
SLC16A12
SLC2A1
SMAD4
SMG9
TBC1D32
TCOF1
TMX3
TOGARAM1
TSC2
TUBB
VAX1
VSX1
WNT2B
ZIC2
ABCA4
ABHD12
ADAM9
ADGRV1
AGBL1
AGK
AGPS
AHI1
ALDH18A1
ALMS1
ALX3
ARL13B
ARL6
ASB10
ATP13A2
BBS1
BBS10
BBS12
BBS2
BBS4
BBS5
BBS7
BBS9

RECQL4
RGS6
RIC1
RNF149
RPE65
RRAGA
RRM2B
RYR1
SALL4
SC5D
SCHIP1
SEC23A
SIL1
SIPA1L3
SIX5
SLC16A12
SLC33A1
SLC40A1
SLC4A4
SLC7A8
SLURP1
SMO
SORD
SOX1
SOX2
SPARC
SRD5A3
STX3
SUOX
TAF1A
TAPT1
TDRD7
TFAP2A
TGFBI
TMCO3
TMEM70
TNPO1
TOR1AIP1
TRAPPC11
TRNT1
TRPM3
TUBA1A
TUBB
TUBB2A
BFSP1
BFSP2
C1QTNF5
C2orf71
C5orf42
C8orf37
CA4
CABP4
CACNA1F
CACNA2D4
CDH23
CDH3
CDHR1
CEP41
CERKL
CHM
CHMP4B
CHST6
CIB2
CLN3
CLN5
CLN6
CLN8
CLRN1
CNGA1
CNGA3
CNGB1
CNGB3
COL11A1
COL11A2
COL8A2
COL9A1
COL9A2
CRX
CRYAB
CRYBA1
CRYGB
CRYGS
CSPP1
CTDP1
CTSD
CYP27A1
CYP4V2
CYP51A1

UCHL1
UNC45B
VCAN
VIM
VLDLR
VSX2
WDR36
WDR87
WFS1
WNT3
WRN
XRCC1
XYLT2
YWHAE
ZNF350

DCN
DDB1
DDB2
DHCR7
DHDDS
DHX38
DPYD
EFEMP1
ELOVL4
ELP4
EP300
EPG5
ERCC2
ERCC3
ERCC4
ERCC5
ERCC6
ERCC8
EYA1
EYS
FADD
FAM111A
FAM126A
FAM161A
FANCA
FANCD2
FANCE
FANCI
FLVCR1
FNBP4
FOXL2
FSCN2
FTL
FYCO1
GALK1
GALT
GCNT2
GFER
GJA3
GNAT1
GNAT2
GNPTG
GPR143
GPR179

GRM6
GRN
GSN
GTF2H5
GUCA1A
GUCA1B
GUCY2D
HARS
HDAC6
HSF4
IDH3B
IKBKG
IMPDH1
IMPG2
INVS
IQCB1
ITPA
ITPR1
JAM3
KAT6B
KCNJ13
KCNV2
KCTD7
KIAA0556
KIF17
KIF26B
KIF7
KLHL7
KRT12
KRT3
LCA5
LCAT
LIM2
LRAT
LRIT3
LRMDA
LZTFL1
MAK
MAN2B1
MERTK
MFN2
MFSD8
MIP
MIR184

MKKS
MKS1
MPLKIP
MTTP
MYH9
MYO7A
NDUFB11
NF2
NOTCH2
NPHP1
NPHP3
NPHP4
NR2E3
NR2F1
NRL
NTF4
NYX
OAT
OCA2
OPA1
OPA3
OPTN
P3H2
PAX3
PCDH15
PDE6A
PDE6B
PDE6C
PDE6G
PDE6H
PDZD7
PEX10
PEX11B
PEX12
PEX13
PEX14
PEX16
PEX19
PEX2
PEX26
PEX3
PEX5
PEX6
PEX7

PHYH
PIKFYVE
PITPNM3
PLA2G5
POLH
POLR1C
POLR1D
PPT1
PRCD
PRDM5
PROM1
PRPF3
PRPF31
PRPF6
PRPF8
PRPH2
RARA
RAX2
RBP3
RD3
RDH12
RDH5
RGR
RGS9
RGS9BP
RHO
RIMS1
RLBP1
ROM1
RP1
RP2
RP9
RPE65
RPGR
RPGRIP1
RS1
SAG
SC5D
SDCCAG8
SEC23A
SEMA3E
SEMA4A
SIL1
SLC24A1

SLC24A5
SLC33A1
SLC45A2
SLC4A11
SNRNP200
SNX3
SPATA13
SPATA7
SPINT2
TACSTD2
TBC1D23
TBX22
TCTN1
TCTN2
TCTN3
TDRD7
TGFBI
TIMM8A
TIMP3
TMEM126A
TMEM138
TMEM231
TOPORS
TP53BP2
TPP1
TRIM32
TRIM44
TRPM1
TSPAN12
TTC21B
TTC8
TULP1
TYR
TYRP1
UBIAD1
UNC119
USH1C
USH1G
USH2A
VCAN
VIM
WDPCP
WDR36
WFS1

WHRN
WNT3
WRAP73
WRN
WT1
XPA
XPC
ZEB1
ZNF408
ZNF423
ZNF513
ARR3
B3GALT1
