# Supplementary material for: The Phenotypic and Genotypic Features of ADAMTSL4‐Related Ocular Disease
Source: Clin Genet. 2025 Nov 17;109(4):730–41. doi: 10.1111/cge.70109 (PMC12958011; doi:10.1111/cge.70109)
Supplement: Supplementary file 2 — Appendix SB: Family pedigrees. [file CGE-109-730-s002.pdf]

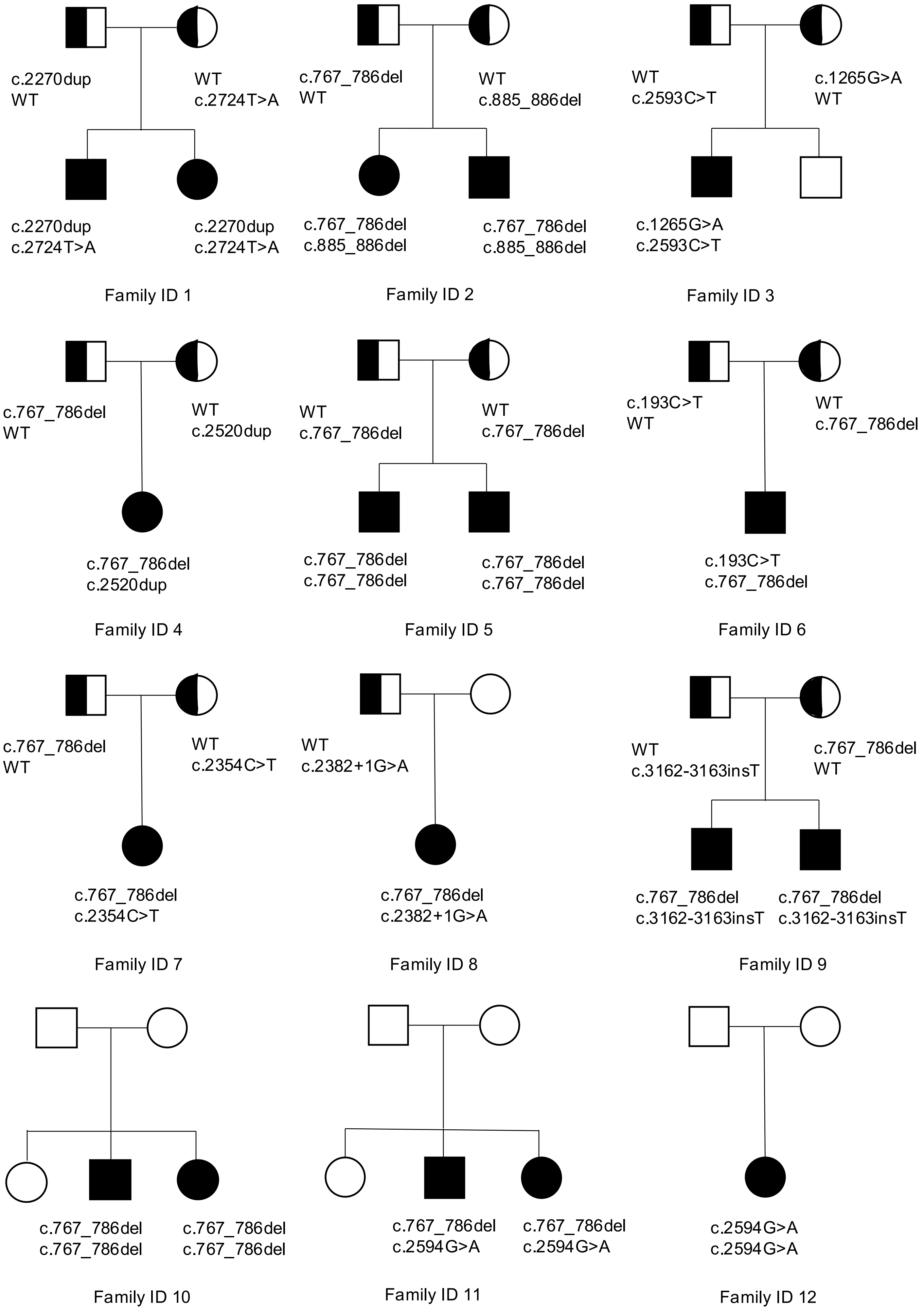
c.2270dup
WT
WT
c.2724T>A
c.2270dup
c.2724T>A
c.2270dup
c.2724T>A
Family ID 1
c.767_786del
WT
WT
c.885_886del
c.767_786del
c.885_886del
c.767_786del
c.885_886del
Family ID 2
WT
c.2593C>T
c.1265G>A
WT
c.1265G>A
c.2593C>T
Family ID 3
c.767_786del
WT
WT
c.2520dup
c.767_786del
c.2520dup
Family ID 4
WT
c.767_786del
WT
c.767_786del
c.767_786del
c.767_786del
c.767_786del
c.767_786del
Family ID 5
c.193C>T
WT
WT
c.767_786del
c.193C>T
c.767_786del
Family ID 6
c.767_786del
WT
WT
c.2354C>T
c.767_786del
c.2354C>T
Family ID 7
WT
c.2382+1G>A
c.767_786del
c.2382+1G>A
Family ID 8
WT
c.3162-3163insT
c.767_786del
WT
c.767_786del
c.3162-3163insT
c.767_786del
c.3162-3163insT
Family ID 9
c.767_786del
c.767_786del
c.767_786del
c.767_786del
Family ID 10
c.767_786del
c.2594G>A
c.767_786del
c.2594G>A
Family ID 11
c.2594G>A
c.2594G>A
Family ID 12

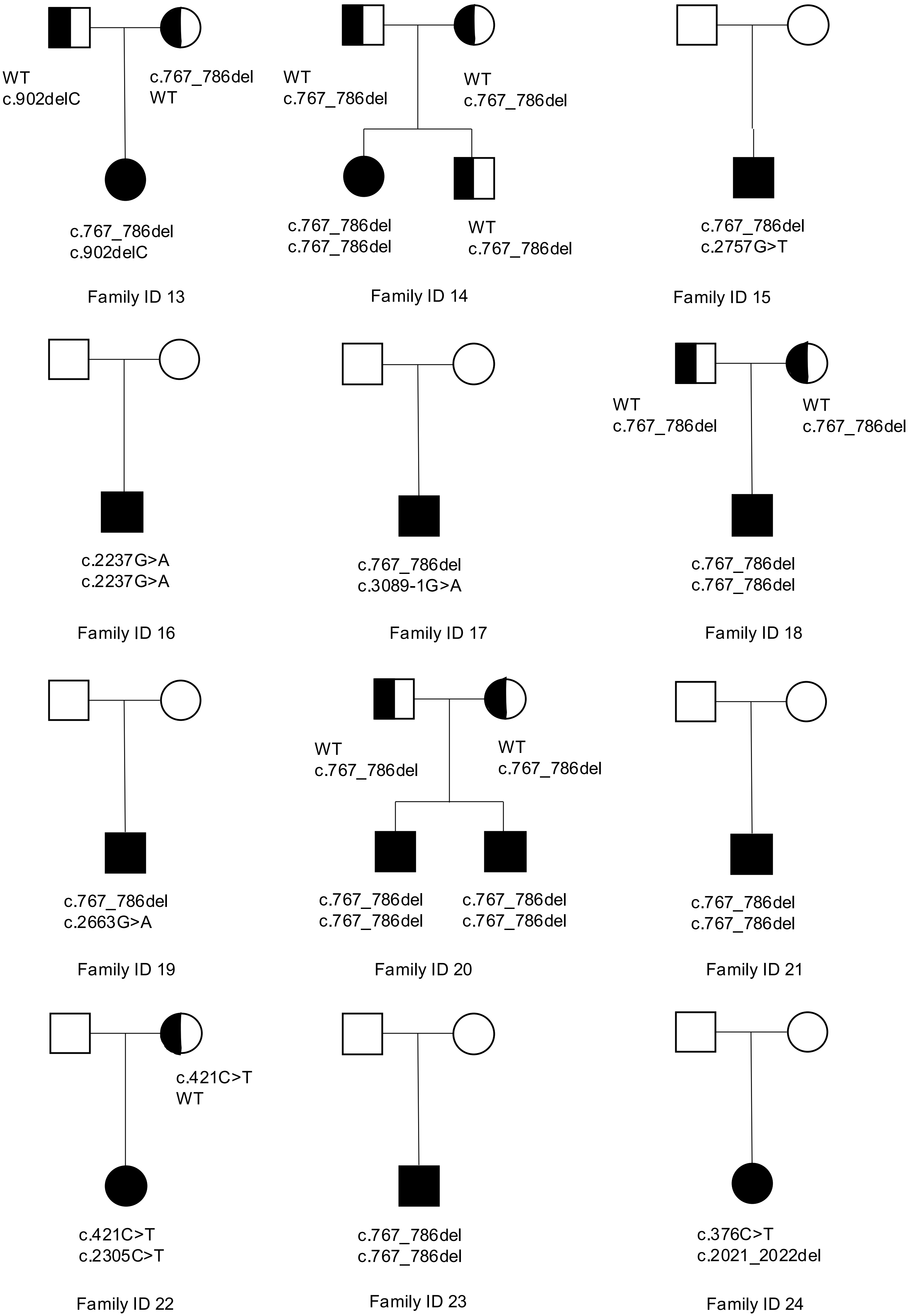
WT
c.902delC
c.767_786del
WT
c.767_786del
c.902delC
Family ID 13
WT
c.767_786del
WT
c.767_786del
c.767_786del
c.767_786del
WT
c.767_786del
Family ID 14
c.767_786del
c.2757G>T
Family ID 15
c.2237G>A
c.2237G>A
Family ID 16
c.767_786del
c.3089-1G>A
Family ID 17
WT
c.767_786del
WT
c.767_786del
c.767_786del
c.767_786del
Family ID 18
c.767_786del
c.2663G>A
Family ID 19
WT
c.767_786del
WT
c.767_786del
c.767_786del
c.767_786del
c.767_786del
c.767_786del
Family ID 20
c.767_786del
c.767_786del
Family ID 21
c.421C>T
WT
c.421C>T
c.2305C>T
Family ID 22
c.767_786del
c.767_786del
Family ID 23
c.376C>T
c.2021_2022del
Family ID 24

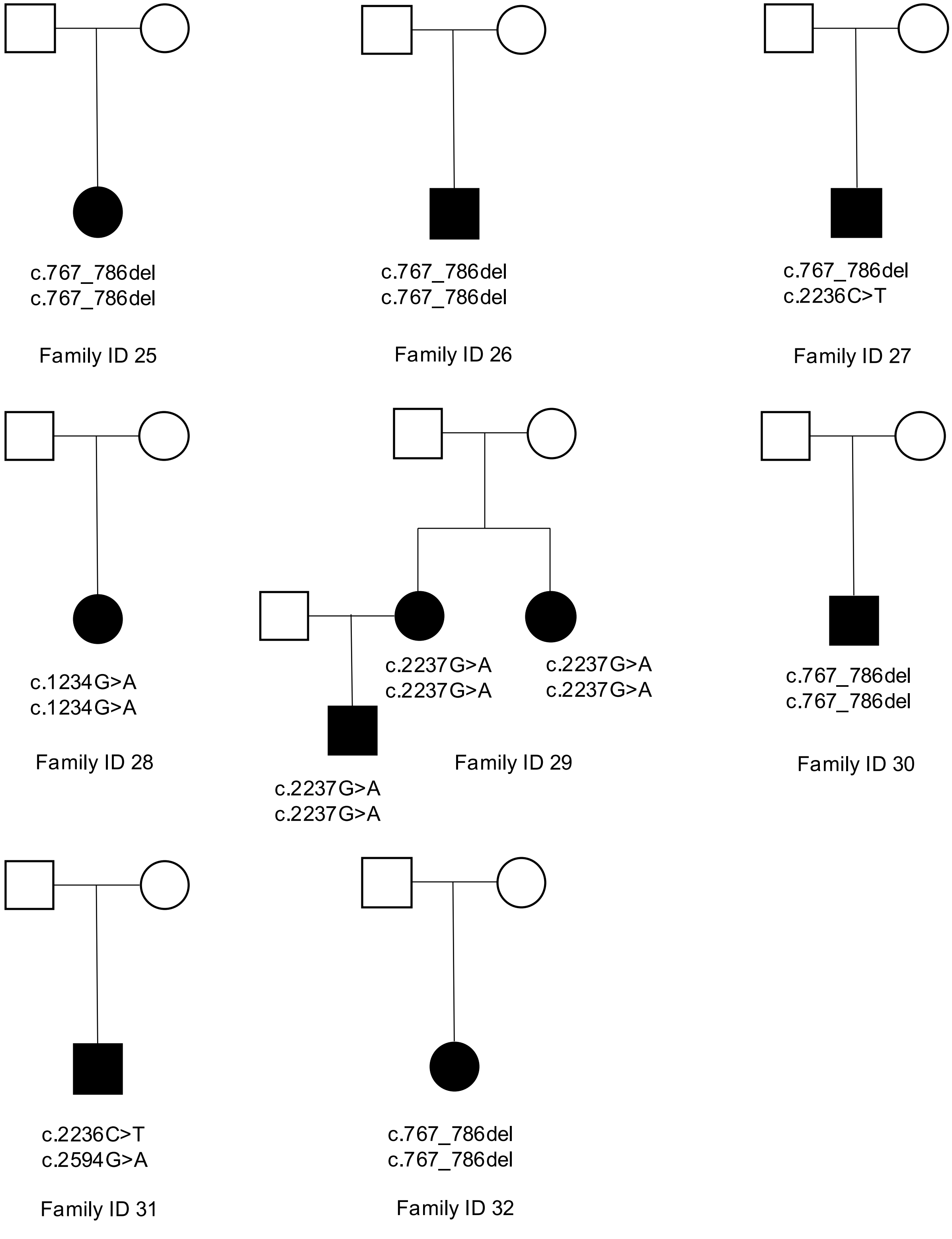

c.767_786del
c.767_786del
Family ID 25
c.767_786del
c.767_786del
Family ID 26
c.767_786del
c.2236C>T
Family ID 27
c.1234G>A
c.1234G>A
Family ID 28
c.2237G>A
c.2237G>A
c.2237G>A
c.2237G>A
c.2237G>A
c.2237G>A
Family ID 29
c.767_786del
c.767_786del
Family ID 30
c.2236C>T
c.2594G>A
Family ID 31
c.767_786del
c.767_786del
Family ID 32
